# Pulmonary and systemic immune alterations in rats exposed to airborne lunar dust

**DOI:** 10.3389/fimmu.2025.1538421

**Published:** 2025-02-06

**Authors:** Brian E. Crucian, Heather Quiriarte, Chiu-wing Lam, Mayra Nelman, Audrie A. Colorado, Douglass M. Diak, John T. James

**Affiliations:** ^1^ Environmental Sciences Branch, NASA Johnson Space Center, Houston, TX, United States; ^2^ School of Kinesiology, Louisiana State University, Baton Rouge, LA, United States; ^3^ Toxicology Laboratory, KBR, Inc., Houston, TX, United States; ^4^ Immunology/Virology Laboratory, KBR, Inc., Houston, TX, United States; ^5^ Immunology/Virology Laboratory, Aegis Aerospace, Inc., Houston, TX, United States

**Keywords:** lunar dust, spaceflight, immunology, gravity, inflammation

## Abstract

**Background:**

Due to cosmic radiation bombardment and over 4 billion meteorite and micrometeoroid impacts on the airless Moon, the lunar surface is covered by a layer of fine, reactive dust. Very little is known regarding the toxicity of lunar dust on human physiology. This study assessed airborne lunar dust exposure in rats on localized pulmonary and systemic immune parameters.

**Methods:**

Rats were exposed to 0 (air only), 20.8 (low), and 60.6 (high) mg/m^3^ of respirable-size lunar dust for 4 weeks (6 h/day, 5 days/week). Rats were then euthanized either 1 day, 7 days, 4 weeks, or 13 weeks after the last exposure. Peripheral blood and lung lavage fluid samples were collected for analysis. Assays included leukocyte distribution by multicolor flow cytometry and electron/fluorescent microscopy to visualize cell–particulate interactions and lavage/plasma cytokine concentration. Mitogen-stimulated cytokine production profiles, as a measure of cellular function, were performed on whole blood samples only.

**Results:**

Untreated lavage fluid was comprised primarily of pulmonary macrophages. High-dose lunar dust inhalation (60.6 mg/m^3^) resulted in an influx of both neutrophils and lymphocytes. Although the percentage of lymphocytes increased, the T-cell CD4:CD8 ratio was unchanged. Cytokine analysis of the lavage fluid showed increased levels of IL-1β and TNFα. These alterations generally persisted through the 13-week sampling. Blood analysis showed few systemic immune alterations from the lunar dust inhalation. By week 4, the peripheral granulocyte percentage was elevated in the treated rats. Plasma cytokine levels were unchanged in all treated rats compared to controls; however, altered mitogen-stimulated cytokine production profiles were observed consisting of increased IL-1β and IL-6 and decreased IL-2. There were minimal adverse immune effects, in both lung or peripheral blood, following low-dose exposure to 20.8 mg/m^3^ lunar dust.

**Conclusion:**

Exposures to high concentrations of lunar dust resulted in persistent lung inflammation and some systemic immune dysregulation that did not subside even 13 weeks after the dust exposure. This information is beneficial in deriving an exposure limit to airborne lunar dust and for spacecraft engineers considering dust mitigation systems in lunar landers or habitats.

## 1 Introduction

Immune system dysregulation, consisting of altered leukocyte distribution, reduced T- and NK-cell function, and low-level inflammation, persists in astronauts for the duration of a 6-month orbital spaceflight (1–3). B-cell function and general hematologic indices appear unaltered in astronauts during spaceflight (4, 5). Suggested causal mission factors include stress, isolation, radiation, microgravity, and circadian misalignment (6). In the same astronaut crew members, elevated levels of latent herpesvirus DNA were detected in saliva, suggesting diminished immunity was associated with virus reactivation (7, 8). Astronauts do experience clinical symptoms during flight, and several case studies of ISS astronauts have been published associating stress and immune compromise with atopic dermatitis and laboratory-confirmed zoster (9–11). It has been suggested that persistent immune dysregulation may increase specific clinical risks for astronaut crew members participating in exploration-class deep space missions (12). As conditions onboard the ISS have continued to improve, and several biomedical countermeasures have been deployed to the ISS, data suggest immunological improvement in more recent crew members (13). Most of these countermeasures do not translate, as currently constituted, to deep space missions, so a specific deep space countermeasure is required (14).

Deep space exploration was recently initiated via the “Artemis” program, planning a return to the Moon to establish an extended human presence. Deployments will include long operations in lunar orbit at the “Gateway” station and landings to extensively explore and inhabit the lunar surface. During Apollo missions, lunar dust was found to pervasively adhere to crew members and lunar landing vehicles, and multiple reports stated this exposure caused notable upper respiratory symptoms in select crew members. It is reasonable to expect that crew immune system dysregulation will worsen during long-duration deep space missions as all the mission stressors will increase in magnitude, habitable volume and power will decrease, and there will be no rapid return option.

Unique surface hazards will include lunar dust as a particulate irritant or allergen. Lunar dust is a unique substance that poses a potential health hazard for lunar surface operations. Due to eons of meteorite bombardment with a complete lack of wind or water erosion, the constant high-speed micrometeoroid bombardments have fractured lunar surface material producing fine dust particles of which approximately 1% to 2% are respirable sizes (≤3 μm). This fraction of fine particles could pose an inhalation hazard to lunar explorers. Trace amounts of virtually all elements ranging from the ppb level to the ppm level can be found in lunar dust (15, 16). However, the bulk chemical composition of lunar dust varies across the lunar surface and is approximately 50% SiO_2_, 15% Al_2_O_3_, 10% CaO, 10% MgO, 5% TiO_2_, and 5%–15% iron, with lesser amounts of sodium, potassium, chromium, and zirconium. The Earth mineral quartz is a standard simulant used by the National Institutes of Health to study respiratory system toxicity as it is known to cause silicosis, and this was used as a positive control in this inhalation exposure study (17).

During exploration missions, human exposure to lunar dust, with the composition as described above, is inevitable regardless of engineering controls (16). Understanding the immune reactivity to lunar dust will provide key knowledge for researchers as we prepare to support prolonged deep space missions. Here, we report our findings in animal exposure studies using lunar dust collected from the Apollo 14 mission relating to immune cell and cytokine responses in both the pulmonary and systemic compartments. Other relevant findings from this extensive animal model study were reported separately (18).

## 2 Methods

### 2.1 Subjects

Subjects for this study were healthy Fischer 344 adult male rats, 8–10 weeks old. Animals were housed in pairs at the AAALAC-approved animal facility at the Johnson Space Center. Subjects were under a controlled environment consisting of HEPA-filtered air and a 12-h day/night cycle. There were 20 rats for each of the four inhalation exposure groups: air control, low-dose lunar dust, and high-dose lunar dust. For each group, a single day of intratracheal quartz treatment was used as a positive control for neutrophilic inflammation. The NASA Institutional Animal Care and Use Committee guidelines were followed.

### 2.2 Lunar dust/quartz exposure

The lunar dust samples consisted of Apollo 14 regolith that had been stored under nitrogen from collection to study initiation. Dust composition and details for this study have been previously disclosed (19). An aerosolized fine fraction was separated using a cyclone and respirable dust was captured on a paper membrane for the inhalation study. Subjects were given a lunar dust inhalation treatment of either low (20.8 ± 2.5 mg/m^3^) or high (60.8 ± 8.1 mg/m^3^) exposure. Quartz used as a control particulate for this study was fine-sized crystalline silica obtained from U.S. Silica (Berkeley Springs, WV). The average size of the quartz particles used for the inhalation study was 0.4 μm. Rats were exposed to sample inhalation for 6 h per day, 5 days per week. Five rats per group were sacrificed and biosamples were collected at each of the following timepoints: +1 day (sampling the next day after exposure), +7 days, +4 weeks, or +13 weeks.

### 2.3 Samples

Biosamples consisted of 1.0 mL of heparin anticoagulated whole blood for immunophenotype, plasma cytokine concentrations and whole blood mitogenic stimulation, and 1.0 mL of peritoneal lavage fluid for immunophenotype and cytokine analysis. Lavage and biosample collection details are previously described (19).

### 2.4 Immunophenotype analysis

To determine cellular composition, flow cytometric analysis was performed on both blood and lavage samples. A multicolor flow cytometry antibody matrix was created that assessed the relevant leukocyte and lymphocyte subsets as follows: granulocytes, lymphocytes, monocytes (CD32 FITC, CD45 PE), B cells, NK cells (CD45RA FITC, CD161a PE), T-cell subsets (CD8 FITC, CD3 PE, CD4 PC5), pulmonary macrophages, dendritic cells, and activated leukocytes (CD11c FITC, CD71 PE). For blood staining, 100 µL of whole blood was then combined with 10 µl of appropriately labeled monoclonal antibodies. Staining was performed by incubating the mixture at room temperature for a minimum of 20 min. Red blood cells were then lysed using the Beckman-Coulter Optilyse reagent as described by the manufacturer, and the cells were washed in phosphate buffered saline (PBS). Lavage staining was performed in a similar fashion, by adding 100 µL of lavage fluid to 10 µL each of the antibodies. After 20 min of incubation at room temperature, the cells were washed in PBS. The stained leukocytes were then fixed in 1.0% paraformaldehyde in PBS for 10 min and analyzed on a Beckman-Coulter Gallios flow cytometer. The cytometer was configured to set the threshold for positive based on isotype control antibody staining or internal negative staining cellular populations. Pure samples of both lunar dust and quartz (the same product administered to the animal subjects) were run to ensure that particulate debris did not interfere with cellular analysis. Electronic compensation was set to eliminate interference due to spectral overlap.

### 2.5 Whole blood cultures

For functional analysis of mitogen-stimulated cytokine profiles, three whole blood cultures were set up by combining 150 µL of heparinized blood and 1.0 mL of RPMI media. Mitogenic stimulation consisted of 0.125 μg/mL of anti-CD3 and 0.25 μg/mL of anti-CD28 soluble antibodies (both from Beckton Dickinson, Franklin Lakes, NJ) to activate T cells only via the T-cell receptor/costimulatory complex, 10 ng/mL of phorbol-12-myristate-13-acetate (PMA) and 2 μg/mL of ionomycin (both from Sigma-Aldrich, St. Louis, MO) as a broader pharmacologic stimulus, or 20 µL of lipopolysaccharide (LPS) to activate the monocytes/macrophages. Cultures were incubated at 37°C for 48 h. Following the culture, the supernatants were removed and frozen until batch analysis.

### 2.6 Soluble cytokine concentration

Concentrations of cytokines were determined in the mitogen-stimulated culture supernatants, plasma, and lavage fluid. The rat Fluorokine MAP MultiAnalyte Profiling immunoassay was performed according to the manufacturer’s instructions (R&D Systems, Minneapolis, MN, USA). This array simultaneously analyzes secreted IFNγ, TNFα, IL-10, IL-6, IL-4, IL-2, and IL-1β using distinct bead populations that fluoresce at different intensities along a single emission wavelength. This assay was performed in a 96-well plate and was analyzed using a Luminex 100 instrument.

### 2.7 Electron microscopy

Electron microscopy analysis was conducted on an XL30 FEI/Philips Environmental Scanning Electron (ESEM) microscope. The environmental mode was used for all imaging. Lung tissue samples were fixed in 2% glutaraldehyde/3% formaldehyde prepared in sterile PBS, pH 7.4, for a minimum of 1 h at room temperature and then stored at 4°C until processing. Lung lavage samples were diluted 1:1 with 4% glutaraldehyde/6% formaldehyde prepared in sterile PBS, pH 7.4, then fixed for a minimum of 1 h at room temperature, and stored at 4°C until processing. Prior to analysis, lung tissue samples were placed into 35 mm dishes (Thermo Fisher Scientific, Waltham, MA, USA), gently rinsed three times with filter sterilized Milli-Q, and then slowly dehydrated (2 changes with each %, 1 h each) with graded alcohol series to 100% ethanol. Samples were placed on double-sided carbon tape mounted on T-stubs and then dried overnight in a dry chamber prior to imaging.

Prior to analysis, lavage samples were placed into filtration units containing 0.05 µm of VmTP Isopore membrane filter (Millipore), gently rinsed three times with filter-sterilized Milli-Q, and then dehydrated with graded alcohol series to 100% ethanol. The VmTP membranes containing the cells were placed on double-sided carbon tape mounted on T-stubs and then dried overnight in a dry chamber. Samples were sputter-coated with gold-palladium prior to imaging. Samples were imaged on a Phillips/FEI Quanta 250 environmental scanning electron microscope (ESEM) with (EDX) under the environmental mode with dual imaging for both secondary (general image morphology) and backscatter modes (highlights elemental variations). Low-magnification imaging was used to acquire large area image mosaics. From these mosaics, regions of interest (ROIs) were identified for subsequent high-resolution imaging and chemical mapping using EDX. Assessment of cellular internalization of particles was carried out by milling selected ROIs with a gallium (Ga)-focused ion beam (FIB) on a Phillips/FEI Quanta 3D field emission gun (FEG) FIB ESEM. Once the cells were selected, a platinum (Pt) protective “bandaid” was layered over the cells followed by milling through the cell regions with the FIB until the cell internal layers were reached, followed by EDX analysis of the internal particles ingested by the cells. Contaminating Pt and Ga peaks are expected due to the layering of the protective Pt coating and milling with the Ga FIB. Remainder peaks are representative of the particle composition inside the cell.

### 2.8 Light microscopy

Conducting light microscopy analysis, lung lavage cells were imaged with an Olympus ×100, 0.95 UMPanF for depth of field illumination. The lavage samples used were similar to the ones fixed for electron microscopy (please see electron microscopy materials and methods). The samples were placed onto a T-stub mount holder and imaged so that the same regions could be imaged using different microscopy techniques and mapped for the identification of cells ingesting particles. Manual 1-µm step images were collected in addition to images in a serpentine pattern with 30% overlap to create an in-focus depth of field mosaic. Final 3D projection images were produced using the Fiji (ImageJ) software package and seamless stitching of the acquired overlapping 3D projections generated with the Hugin software package. Light microscopy analysis of the lung tissues was performed on a Keyence VHX-600 microscope. The lung tissues used were similar to the ones fixed for electron microscopy (please see electron microscopy materials and methods). The samples were placed onto a T-stub mount holder and imaged with a full illumination cap on a ×20–×200 objective; images were collected at ×75 magnification. Manual 1-µm step images (4,800 × 3,600) were collected from the base of the tissue to the top, with the final 3D projection produced using the Fiji (ImageJ) software package. Large tissue sections requiring stitching several 3D projections were generated using the Hugin software package.

### 2.9 Statistical analysis

Statistical significance was evaluated for air control and low- and high-dose lunar dust using a two-way ANOVA (treatment and time) for each analyte tested. Multiple comparisons (Dunnett) were analyzed for the air control at each timepoint (dose) and for each group’s day 1 value (time). Quartz was not included in the analysis as it was used as a positive control to ensure assay quality (data not shown but provided upon request). The mean differences between time course and treatment type were considered significant if *p <*0.05.

## 3 Results

### 3.1 Microscopy analysis

**Figure 1:**
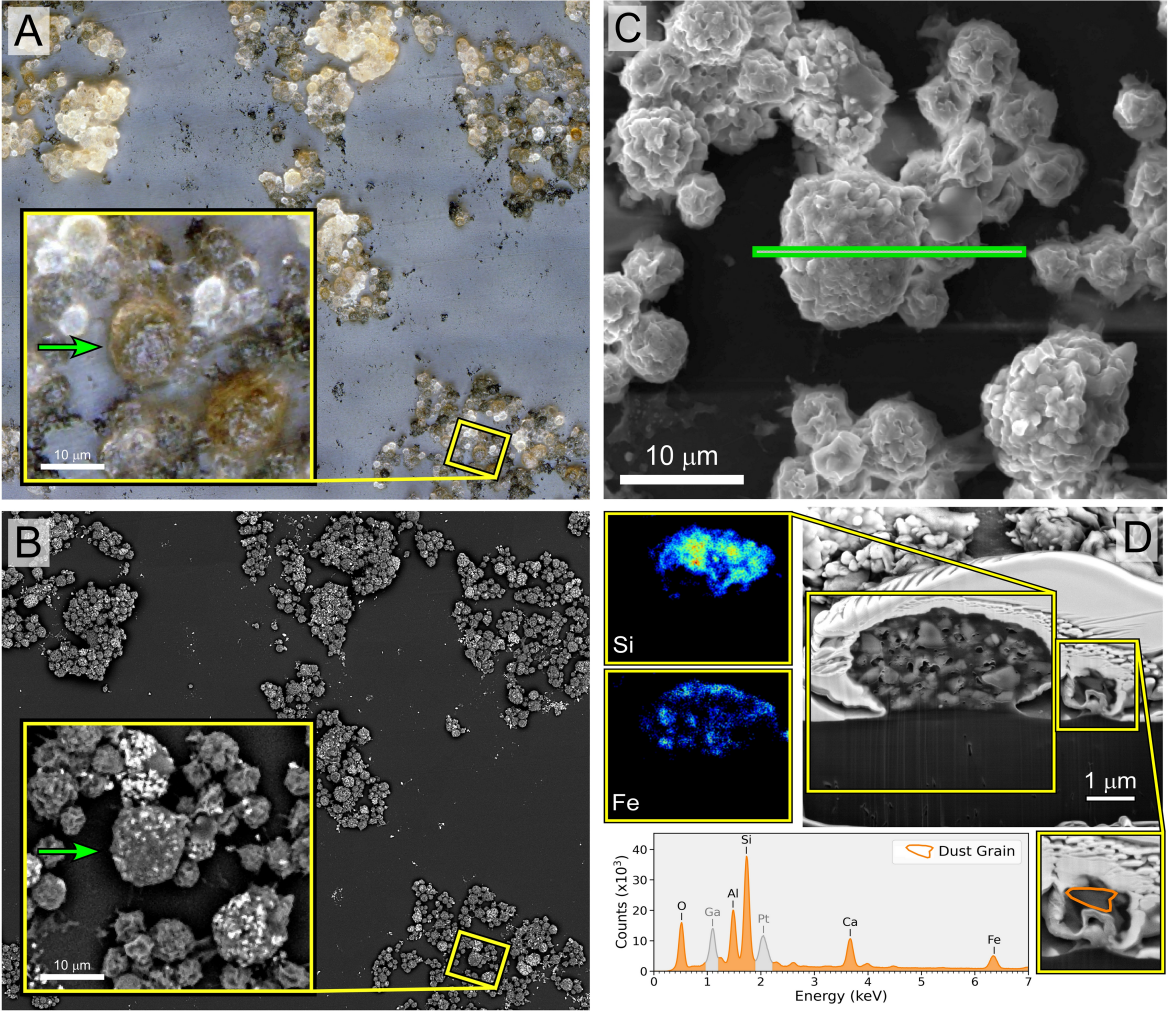
Image panel of macrophage/neutrophil cells isolated from the rat lunar dust inhalation study lung lavage samples from a rat exposed to high lunar dust loads for 13 weeks. The application of different methods of imaging to the same region enabled the identification of cells that have ingested lunar particles for confirmation by EDX. **(A)** Extended depth-of-field image of a lung lavage sample with visible lunar dust (black debris) and macrophage and neutrophil cells that ingested lunar dust particles (brown and black cells). **(B)** Backscatter electron microscopy imaging of the same region, highlighting elemental variations of the lunar dust on the outside of the cells (bright particles). **(C)** The cross-section of the region of interest for focused ion beam (FIB) sectioning of both macrophage and neutrophils that were identified in **(A)** to have ingested particles. **(D)** The cross-section of the two cell types along with EDX analysis of the macrophage interior showing the presence of Si and Fe confirming the presence of lunar dust.

Light microscopy analysis of the lavage samples confirmed the presence of both leukocytes and lunar dust (**Figures 1A, B**). Focused ion beam sectioning indicated that phagocytic cells did ingest lunar dust particles, confirmed by elemental analysis (**Figures 1C, D**).

### 3.2 Control cytometry

**Figure 2:**
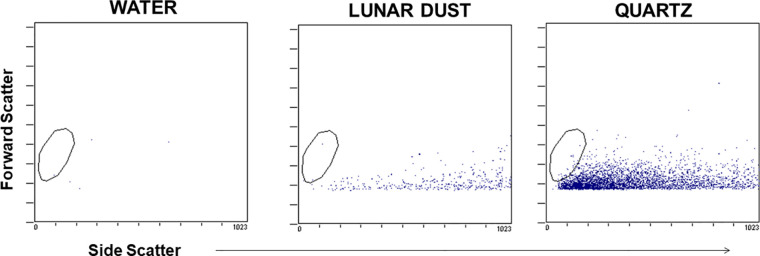
Representative flow cytometry dot plots demonstrate that the optical scatter properties for the lunar dust and quartz inhalation mixtures did not overlap with the cellular analysis areas (typical lymphocyte size indicated by the visible gate region).

Flow cytometric analysis of both lunar dust and quartz alone confirmed that these particles did not possess either forward- or side-light scatter properties which would interfere with the cellular analysis of the lavage fluid (**Figure 2**). Both particles possessed forward-scatter properties below that expected for normal lymphocyte size (~10 μm), which would likely be the smallest expected cell population of interest within either the lavage or blood samples. Lunar dust appeared to possess higher side-scatter properties, whereas the quartz particles possessed lower side-scatter properties.

### 3.3 Lavage cytometry

**Figure 3:**
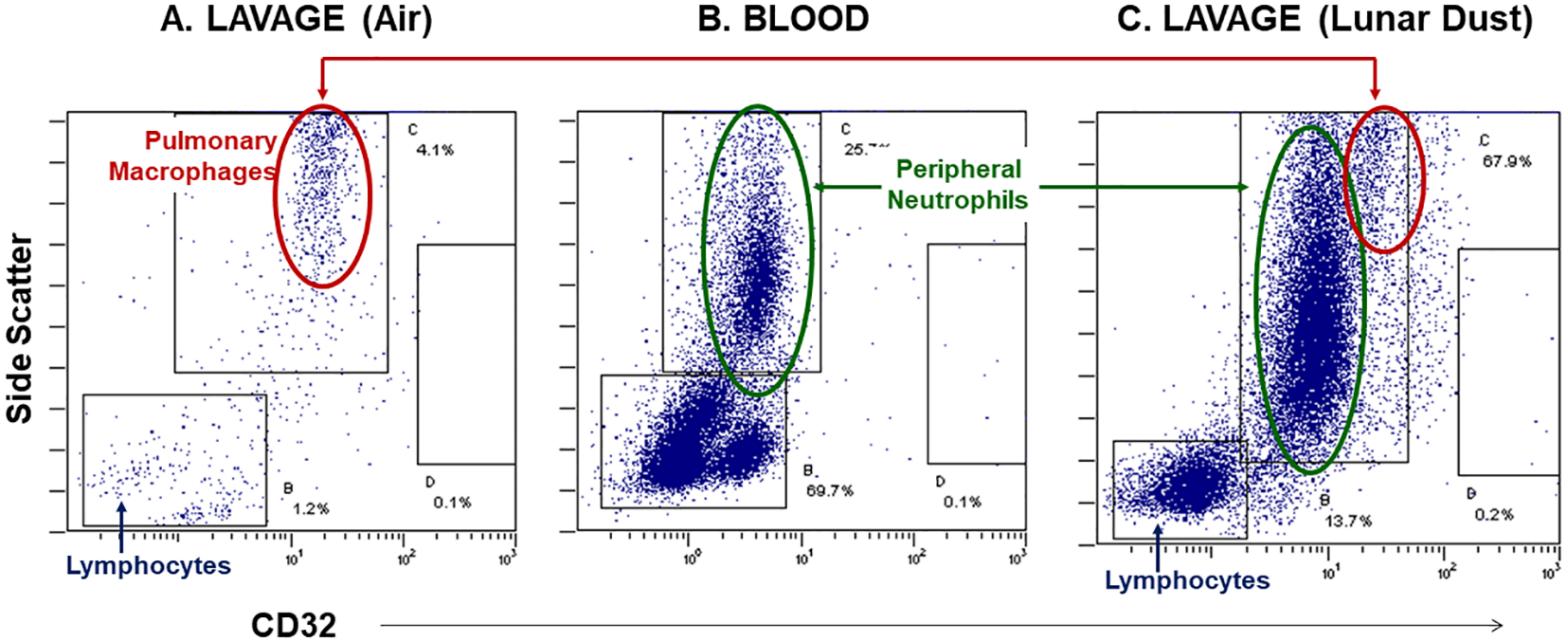
Representative flow cytometry dot plots, side-scatter vs. CD32 expression, illustrate the neutrophil influx into the peritoneal space following lunar dust inhalation. **(A)** Control lavage consists primarily of pulmonary macrophages; **(B)** rat blood staining illustrates the location of peripheral granulocytes; **(C)** the treated lavage fluid consists of both macrophages and neutrophils, with an increased population of lymphocytes.

**Figure 4:**
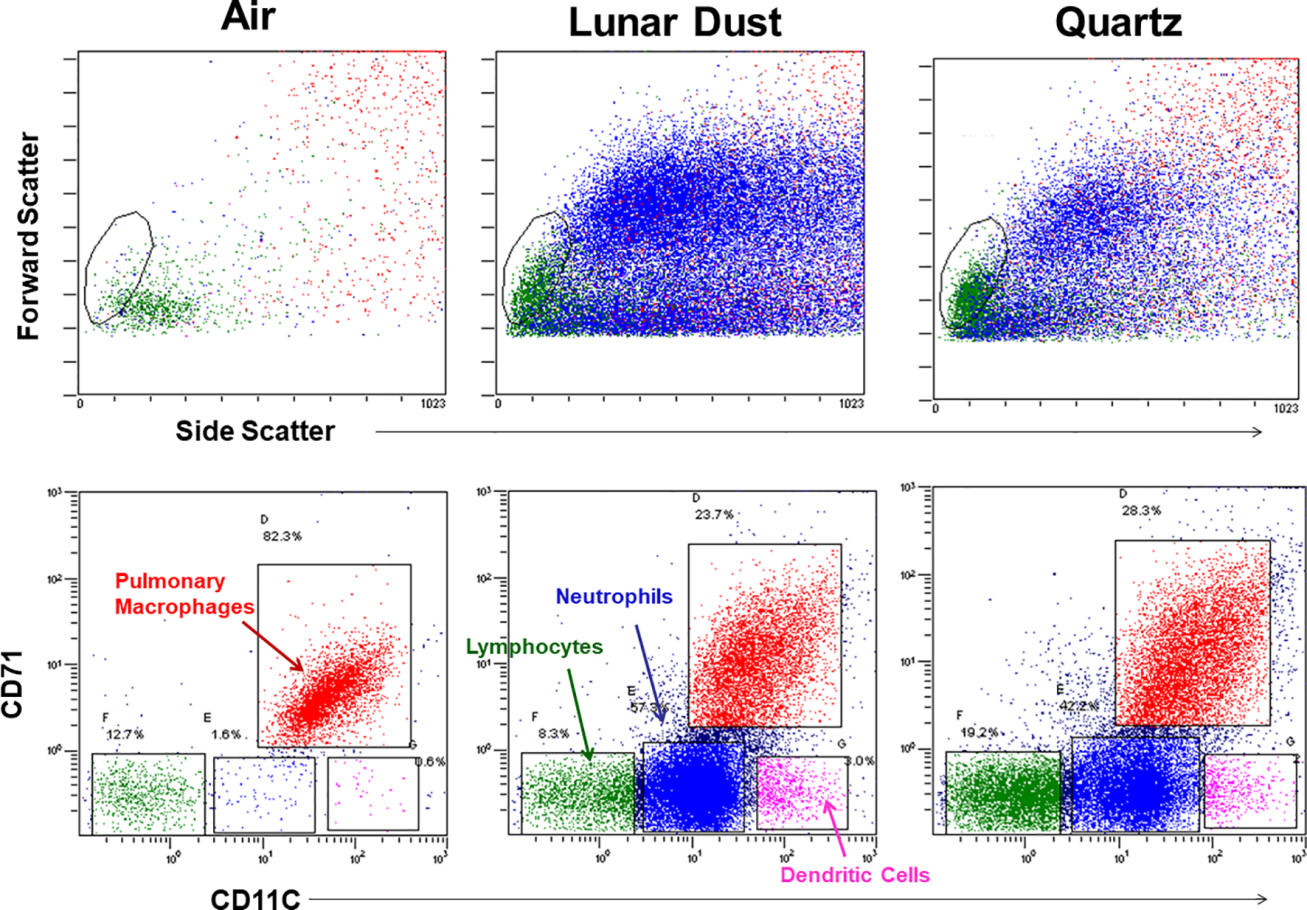
Flow cytometry cellular characterization of pulmonary lavage from air-, lunar dust-, and quartz-treated representative subjects based on scatter properties and expression of CD71 and CD11c. The air-treated normal lavage fluid consists primarily of CD11c^+^/CD71^+^ pulmonary macrophages. After lunar dust and quartz inhalation treatment, a pulmonary influx of neutrophils, dendritic cells, and lymphocytes is evident.

After identifying and gating all leukocytes via CD45 expression, based on CD32 expression and scatter characteristics, it was possible to resolve normal pulmonary macrophage and lymphocyte populations by flow cytometric analysis of lavage samples from air-treated subjects (**Figure 3**). A similar analysis of rat peripheral blood revealed a primary composition consisting of neutrophils and lymphocyte subsets (**Figure 3**). Analysis of lavage samples from lunar dust-treated animals demonstrated the influx of neutrophils and lymphocytes into the lungs (**Figure 3**). Lavage fluid was further analyzed based on the expression of both CD71 and CD11c (ungated to resolve all populations) to confirm the phenotype of the pulmonary macrophage population (CD71^+^CD11c^+^) and quantify the relative percentage of macrophages, lymphocytes, neutrophils, and dendritic cells in all samples. The analysis strategy with the identification of the leukocyte populations for the representative air, lunar dust, and quartz lavage samples is shown in **Figure 4**.

### 3.4 Lavage leukocyte distribution

**Figure 5:**
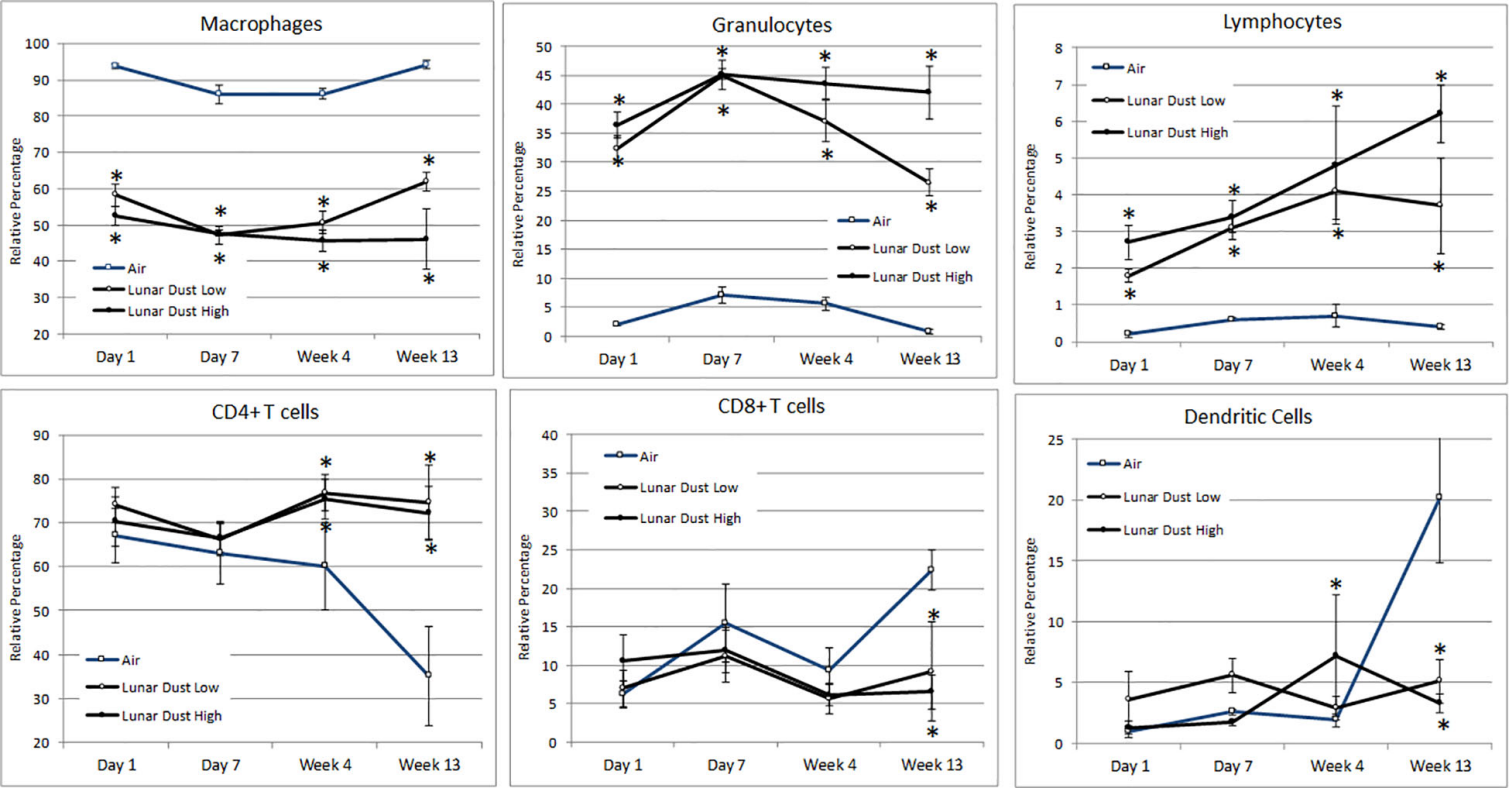
Mean alterations in lavage leukocyte distribution by inhalation treatment and time course. Data are expressed as relative percentage mean ± SEM for the indicated parameters. Lunar dust data, high and low concentrations, were evaluated for significance at each timepoint against the corresponding “air control” data point (“*,” *p* ≤ 0.05).

Flow cytometric analysis of the air-treated lavage samples confirmed that the normal lavage (air-treated, day 1) consisted of mostly pulmonary macrophages (>90%), with a small percentage of lymphocytes and granulocytes also present (**Figure 5**). Among the lymphocytes present, the majority were CD4^+^ T cells and not CD8^+^ T cells (**Figure 5**). Following treatment with either lunar dust (low or high concentration) or quartz, the lavage cellular composition changed over time primarily due to a significant influx of granulocytes and lymphocytes (**Figure 5**). Although inconsistent across the timepoints, it appears that the lymphocytic infiltrate consisted of CD4^+^ T cells, significant at the 4- and 13-week timepoints, as compared to the air-treated control samples. This shift in CD4^+^ T cells also coincided with a lower relative percentage of CD8^+^ T cells in the low- and high-dose lunar dust groups at week 13 (**Figure 5**). Dendritic cell percentages in lavage fluid were variable between the low-dose and high-dose groups, with higher lunar dust concentrations yielding a significant increase at week 4 in dendritic cell percentage, whereas both the low and high doses were actually significantly reduced at week 13 compared to air controls (**Figure 5**).

### 3.5 Lavage cytokine levels

**Figure 6:**
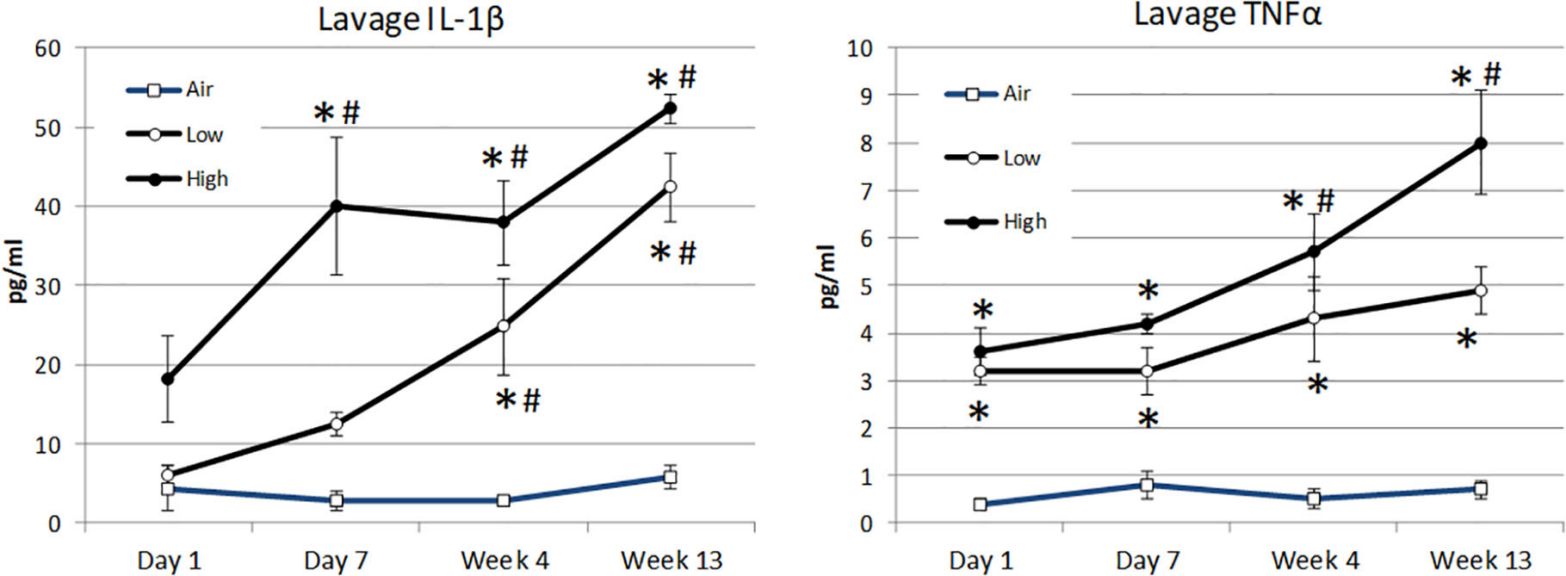
Mean alterations in lavage cytokine concentration of IL-1β **(A)** and TNFα **(B)** by inhalation treatment and time course. Data are expressed as mean pg/mL cytokine concentration ± SEM for the indicated analytes. Lunar dust data, high and low concentrations, were evaluated for significance at each timepoint against the corresponding “air control” data point (“*,” *p* ≤ 0.05). Significance was also evaluated over time, comparing day 7, week 4, and week 13 to the day 1 timepoint (“#”).

Although an array of seven cytokines was assessed in the subjects’ lavage fluid, statistically significant differences were only observed following inhalation in the concentration of IL-1β and TNFα. Compared to the air-treated control subjects, the concentration of IL-1β was significantly elevated by day +7 for high-dose lunar dust. By 4 weeks, both low- and high-dose lunar dust were significantly high, and the concentration continued to increase through 13 weeks of treatment (**Figure 6**). Additionally, IL-1β was significantly increased in the low lunar dust-treated subjects at week 4 and week 13 compared to their retrospective day 1 subjects. Within the high-dose lunar dust groups, all three timepoints (day 7, week 4, and week 13) were significantly increased in IL-1β compared to day 1.

The lavage concentration of TNFα was significantly elevated in all treatment groups at day 1 as compared to the air-treated control subjects and continued to be significant at each timepoint thereafter (**Figure 6**). Similar to IL-1β, the concentration of TNFα continued to rise for all treatment groups through week 13; however, this was only significantly increased in the high-dose lunar dust group at weeks 4 and 13. The low-dose lunar dust group did not reach statistical significance (day 1 vs. week 13; −1.74 pg/mL, *p* = 0.07).

### 3.6 Blood immune alterations

**Figure 7:**
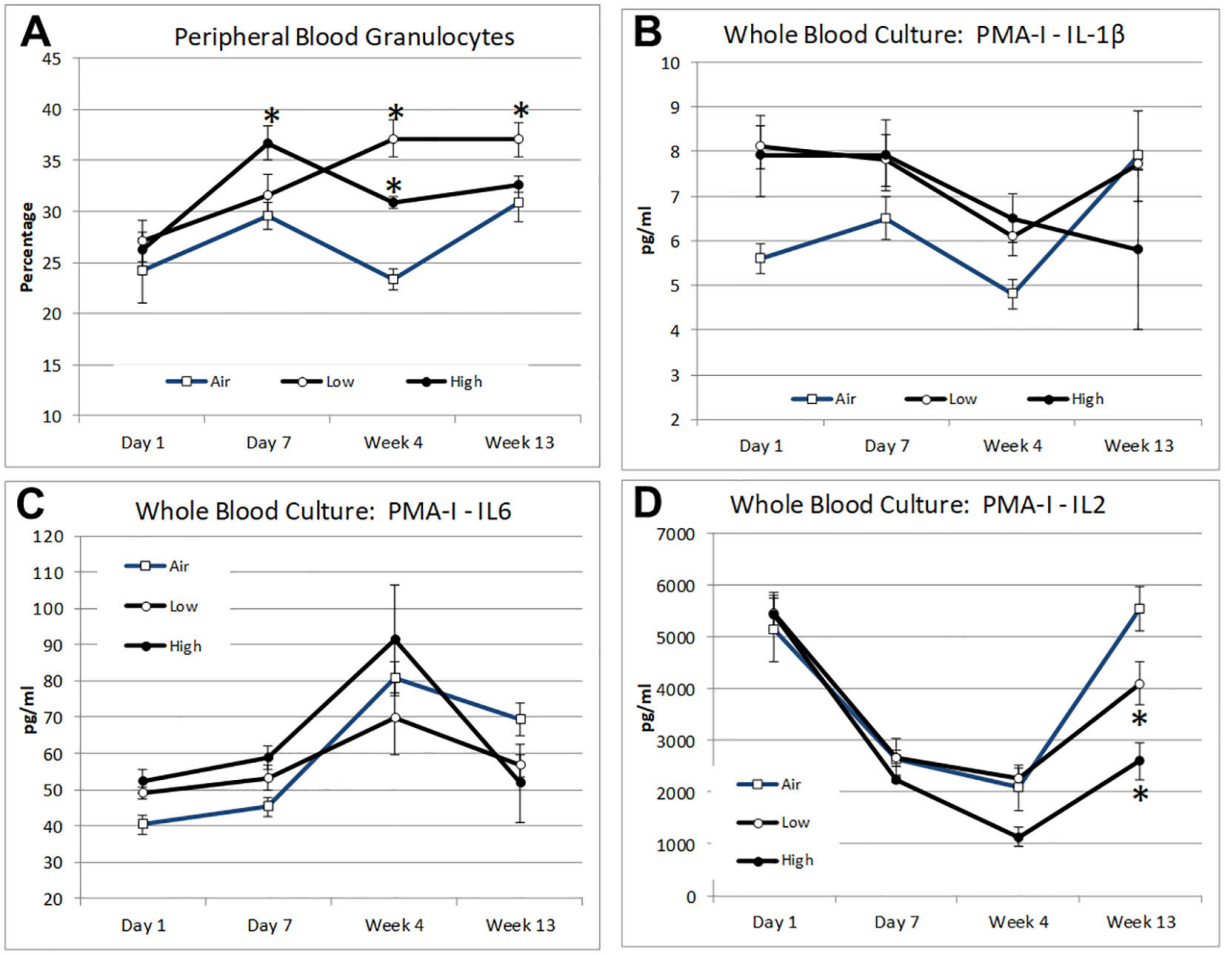
Mean alterations in peripheral blood leukocyte distribution and mitogen-stimulated cytokine response by inhalation treatment and time course for peripheral blood granulocytes **(A)**, whole blood treated with PMA + ionomycin IL-1β **(B)**, whole blood treated with PMA + ionomycin IL-6 **(C)**, and whole blood treated with PMA + ionomycin IL-2 **(D)**. Data are expressed as mean (relative percentage or pg/mL concentration as indicated) ± SEM for the indicated parameters. Lunar dust data, high and low concentrations, were evaluated for significance at each timepoint against the corresponding “air control” data point (“*,” *p* ≤ 0.05).

The same leukocyte subset analysis as used for the lavage samples was also performed for the peripheral blood samples from each subject. Compared to the air-treated control subjects, a significant increase in the peripheral blood granulocyte percentage was observed in the lunar dust-treated subjects at day 7 and week 4, for the high-dose subjects, and at weeks 4 and 13, for the low-dose subjects (**Figure 7**). A concurrent significant decrease in the peripheral lymphocyte percentage was observed for the lunar dust-treated subjects (both doses) only at the week 4 timepoint (data not shown). Within the lymphocyte subsets, CD4^+^ T cells in the high-dose lunar dust group did rise significantly at day 7 compared to their corresponding day 1 values, but this leveled off by week 4. Activated leukocytes in the bloodstream were lower in the lunar dust groups compared to the air-treated control. However, this was only significantly lower at day 7 for the high-dose lunar dust group (data not shown). Additionally, this drop in activated leukocytes at day 7 for the high-dose lunar dust group was also significantly lower than the high-dose lunar dust group at day 1. There were no other consistent significant differences observed in the peripheral blood leukocyte subsets for any subjects.

Plasma cytokine levels were assessed for all subjects, using the same seven-cytokine array as was used for the lavage analysis. Opposite to the lavage fluid data, IL-1β was significantly lower in the high-dose lunar dust group compared to the air-treated controls at day 1 (data not shown). Additionally, IL-1β was significantly higher in the low-dose lunar dust group compared to the air-treated controls at day 7. IL-1β leveled out to the same as the controls at week 4 and beyond. The concentrations for all cytokines measured were unchanged within all treated groups, at all timepoints, compared to the air-treated control subjects (data not shown).

As a measure of leukocyte functional capability, the supernatant cytokine levels were also assessed from mitogen-stimulated whole blood cultures. Following lunar dust treatment, no significant differences were observed in cytokine production following T-cell stimulation (antibodies to CD3 and CD28) or following LPS stimulation (data not shown). Following stimulation in the presence of PMA + ionomycin, as a more powerful pharmacologic stimulus, there were significant differences observed in both the lunar dust doses as compared to the air-treated control with a decreased production of IL-2 (week 13) (**Figure 7**). While not statistically significant, there was a notable increase of IL-1β following PMA+I stimulation in both the low- (+2.44 pg/mL; *p* = 0.05) and high-dose lunar dust (+2.24 pg/mL; *p* = 0.08) groups compared to the air-treated control at day 1. There were no other consistent significant differences observed in the functional capacity of peripheral blood leukocytes in response to PMA + ionomycin.

## 4 Discussion

Immune system dysregulation involved with spaceflight has been well documented by researchers, and we can only expect this to worsen with future deep space, longer-duration missions (12). Recent data have confirmed that this phenomenon occurs during short- and long-duration spaceflight, indicating an in-flight occurrence, not merely a post-flight landing effect (2, 20). T cell, natural killer (NK) cell, monocyte, and neutrophil function are all found to be diminished following spaceflight (1–3). The reactivation of latent herpesviruses, likely as a result of diminished immune control, has also been documented to occur during spaceflight (7, 8). There have been Apollo program reports of lunar dust exposure leading to notable upper respiratory symptoms in select crew members (21, 22). As human exploration of the Moon may soon resume, the immunoreactivity of lunar dust must be considered as frequent lunar surface operations will be conducted to begin building for mankind’s long-term presence in deep space (15).

Lunar dust is a unique substance that poses a potential health hazard to crew members that may already be experiencing a decrease in immune function. Most studies performed in other animal and *in-vitro* models use simulant, as true lunar regolith is federally controlled, and to obtain samples, laboratories must go through a lengthy approval process. The current study is novel and uniquely designed to assess actual lunar dust immune-reactivity. As such, our laboratory applied and was approved to obtain lunar dust from the Apollo 14 mission. This was a large and interdisciplinary rodent model investigation. The parent investigation thoroughly investigated the toxicity of lunar dust in the same rat inhalation model, and the overall relevant findings, including the bulk characterization of neutrophils and lymphocytes in blood and lung tissue, were previously reported (18). This current immune-centric substudy examined immunotoxicity and reactivity, specifically in lung lavage fluid and peripheral blood leukocyte distribution and cytokine responses.

Lunar dust is comprised of micron-sized sharp particles, and the bulk chemical composition of lunar dust varies across the lunar surface (15). For this study, we used the Earth mineral quartz as a standard simulant commonly used by the National Institutes of Health to study respiratory system toxicity as it is known to cause silicosis (17). We confirmed that the optical scatter properties for the lunar dust inhalation or quartz treatment mixtures did not interfere with the flow cytometric cellular analysis (**Figure 2**) and that it was possible to resolve normal pulmonary macrophage and lymphocyte populations by flow cytometric analysis of lavage samples from air-treated subjects (**Figures 3**, **4**). Our study showed an influx of neutrophils, dendritic cells, and lymphocytes, primarily CD4^+^ T cells, into the lungs of animals treated with lunar dust and quartz inhalation (**Figure 5**). A reduction in dendritic cells was observed after 13 weeks of lunar dust treatment (both doses); however, this finding appeared to be artifactual and resulted from an increase in the air control-treated subjects (**Figure 5**). Within the lavage fluid, cytokine analysis showed only that IL-1β and TNFα were significantly increased after treatment with lunar dust and quartz by day 7 through week 13 (**Figure 6**).

The same analysis was conducted in the peripheral blood samples and showed an increase in the blood granulocyte population in the lunar dust-treated animals. The only significant differences observed in plasma cytokine levels were after PMA + ionomycin stimulation in the high-dose lunar dust concentration treatments including increased IL-1β and IL-6 and decreased IL-12 (**Figure 7**).

The findings from the current investigation are in relative alignment with the observations terrestrially of lung inflammation resulting from particulate inhalation (23) and from the parent studies’ high-level assessment of bulk lymphocyte subsets in conjunction with other biomarkers (18). Lam reported concentration-dependent increases, as detected by the simple Wright stain and manual differential analysis, in the levels of lung neutrophils and lymphocytes but no increase in the total cellular concentration. This likely reflects the influx of the neutrophils and lymphocytes in response to the particulate and only a relative decrease in pulmonary macrophages, as this method does not quantify absolute concentrations of cells (only relative percentages). The current study adds to this dataset via positively identifying populations by surface marker expression and by adding a higher resolution snapshot of leukocyte distribution assessing additional populations (T-cell subsets, etc.). The influx of lymphocytes is revealed to be primarily CD4^+^ T cells, also in accordance with the expectations for a particulate pulmonary insult.

This work has implications for ensuring crew safety during the upcoming “Artemis” missions to the lunar surface. Lunar dust as a human health hazard has been known since the Apollo missions, which found the dust to be extremely adherent to surfaces and clothing and became somewhat pervasive within the crew cabin. A lack of gravity (or 1/6 reduced gravity) only serves to worsen the exposure as small particles do not settle out readily over time. These challenges must be addressed through appropriate operational (e.g., cleaning) and engineering controls (e.g., high-efficiency particulate air filtration), and these mitigations must be informed by health-based guidelines to adequately protect crew health. Compounding the immune crew health risk are the other stressors associated with lunar exploration, including elevated radiation exposure, the stress associated with confinement and isolation, and circadian misalignment. In synergy, these combined effects may increase the hazard over the effects that might be observed during a terrestrial exposure. For example, onboard the ISS, several case studies have documented that the persistent immune system dysregulation in astronauts has, in selected crew members, been associated with adverse clinical outcomes. These included atopic dermatitis, atypical allergy, and in a recent case, laboratory-confirmed zoster. These data are relevant therefore to understanding the true clinical risk associated with lunar dust inhalation, as they establish “baseline consequences”.

We acknowledge several limitations of this study, including a focus on animal models. Care must be taken in extrapolating rodent data to human clinical risk, but these data represent a first step in understanding that risk. We suggest that future studies assess the effect of lunar dust exposure in similarly immune-compromised subjects. These could include cell culture-modeled microgravity studies, additional rat studies using hindlimb unloading, or even human documentation of occupational exposures in extreme environments such as undersea or military deployment or during Antarctica winterover. Indeed, in a submitted companion article to this work, we disclose the results from a recent human primary immune cell culture experiment also using actual lunar dust samples, to assess both immediate and inducible “allergic” responses (data not shown). The goal regarding all mission-associated stressors is mitigation. Recent progress has been made toward a biomedical countermeasure to rectify immune competence for deep space missions. This includes the identification of countermeasure options (14) and validation of perhaps the most appropriate human mission analog platform for countermeasure validation (24). Recently, NASA has initiated a study to assess the effectiveness of a specific targeted multisystem biomedical countermeasure during Antarctica winterover deployment. We further suggest that the “challenge” of immune cells from such individuals may be informative regarding how sensitivity to lunar dust evolves during an immune compromise situation, or the even post-flight challenge of the first Artemis crew members, the first humans since Apollo to have a true “occupational exposure” to actual lunar dust, will also be informative. Taken all together, the rodent and human study data could be expanded and greatly facilitate our understanding and mitigation of lunar dust risk for the upcoming Artemis missions.

## Data Availability

The raw data supporting the conclusions of this article will be made available upon request to the NASA Life Sciences Data Archive.
